# Cross-sectional study of the association between day-to-day home blood pressure variability and visceral fat area measured using the dual impedance method

**DOI:** 10.1371/journal.pone.0206945

**Published:** 2018-11-05

**Authors:** Junko Kuwabara, Koichiro Kuwahara, Yoshihiro Kuwabara, Shinji Yasuno, Yasuaki Nakagawa, Kenji Ueshima, Takeshi Kimura

**Affiliations:** 1 Department of Cardiovascular Medicine, Kyoto University Graduate School of Medicine, Kyoto, Japan; 2 Department of Cardiovascular Medicine, Shinshu University School of Medicine, Matsumoto, Japan; 3 Center for Accessing Early Promising Treatment, Kyoto University Hospital, Kyoto, Japan; 4 Department of EBM Research, Institute for Advancement of Clinical and Translational Science, Kyoto University Hospital, Kyoto, Japan; International University of Health and Welfare, School of Medicine, JAPAN

## Abstract

**Background:**

The blood pressure (BP) variability (BPV) is a predictor of cardiovascular disease, independently of the BP itself. In addition, visceral fat accumulation can trigger atherosclerotic disease through various mechanisms.

**Methods and results:**

We examined the association between fat accumulation and day-to-day BPV in 61 adult hypertensive patients. Visceral fat area (VFA) was measured using the dual bioelectrical impedance analysis method. Participants were divided into three groups based on VFA. The standard deviation (SD) in home systolic BP (SBP) for 7 consecutive days was significantly lower in the high VFA tertile (low VFA, 8.40±4.15 mmHg; intermediate VFA, 8.47±2.80 mmHg; and high VFA, 5.84±2.37 mmHg, p of One-way ANOVA = 0.017, p for trend = 0.0126). A similar association was observed between the coefficient of variance (CV) of home SBP and the VFA tertile. Multiple-regression analysis adjusted for age, sex, antihypertensive drug, diabetes, habitual drinking, and SBP level also showed a significant association between the VFA tertile and the SD or CV of home SBP. The adjusted coefficient of regression for the SD of home SBP was -3.28 (95%CI: -5.60 to -0.97, p = 0.008) and the CV of home SBP was -2.51 (95%CI: -4.31 to -0.71, p = 0.008) for the highest VFA tertile as compared to the lowest VFA tertile.

**Conclusions:**

These results show for the first time negative correlation between VFA and day-to-day BPV. The degree of obesity should be taken into account when evaluating the value of BPV.

## Introduction

It is now recognized that for management of hypertension, blood pressure (BP) measured at home provides more reliable information than that measured in the doctor’s office, as it avoids observer bias, regression dilution bias and the white coat effect [1]. Indeed home BP measurement offers better prognostic significance and is more indicative of target organ damage than office BP measurement [2]. Consequently, home BP measurement is recommended in current guidelines and is widely practiced in developed countries [3,4].

The clinical significance of home BP measurement is attributed to multiple measurements, which can include both short and long-term BP variability (BPV). This is noteworthy because in addition to absolute BP levels, themselves, the variability in the BP is associated with adverse cardiovascular consequences [5]. Indeed, it was recently reported that day-to-day BPV is independently predictive of cardiovascular outcomes [6–9]. However, the available information about the determinants that affect day-to-day BP variability remain limited.

Obesity is known to be one of the major causes of hypertension. Sympathetic activation contributes to the development of hypertension in obese patients via direct and indirect effects including renin-angiotensin-aldosterone system (RAAS) stimulation [10]. However, the relationship between obesity and day-to-day BP variability is not well understood, though there is one report showing an inverse correlation between body mass index (BMI) and day-to-day BP variability at home [11].

Although BMI reflects visceral fat accumulation, BMI is also not perfect as a parameter of visceral fat accumulation because it is also affected by accumulation of subcutaneous fat and muscle mass. Indeed, It was recently suggested that the associations between BMI, percent body fat, and health risks differ between Asian and European populations [12,13]. For instance, visceral fat area (VFA) is reportedly larger in Japanese than Caucasians after adjusting for subcutaneous fat area, age and sex [13], and average BMI is significantly lower in Japanese diabetic patients (23.1 kg/m2) than patients in the UK (29.4 kg/m2) [12]. In the present study, therefore, we focused on the association between day-to-day BP variability at home and visceral fat accumulation estimated using a newly developed device that can accurately measure visceral fat area without X-ray exposure.

## Methods

### Study design and participant population

This study is a single-center, cross-sectional study to examine the association between day-to-day BPV and fat accumulation. For this study, adults aged 20 years or older with diagnosed hypertension were consecutively recruited from the outpatient department of the Kyoto Station Horii Medical Clinic in Kyoto, Japan. Because this is an exploratory study and no study evaluating the association between VFA and BPV had not been reported, we determined the sample numbers to be 100 cases in consideration of feasibility of the study. Hypertension was defined as an office BP greater than or equal to 140/90 mmHg or a home BP greater than or equal to 135/85, or as receiving anti-hypertensive treatment in accordance with the guidelines of the world at the time [14][15]. There were no exclusion criteria, and untreated hypertensive patients were also eligible for the study. A total of 102 patients with consent were enrolled between April 2013 and September 2014. Forty patients who have only BP records on less than 5 days within consecutive 7 days and one patient who failed to measure visceral fat area were excluded based on the protocol. Finally 61 subjects were analyzed. Baseline characteristics data including age, sex, medication, medical history and drinking habit were obtained medical records and interviews with participants. This study protocol was consistent with the ethical guidelines of the 1975 Declaration of Helsinki and approved by the Ethics Committee of Kyoto University Graduate School of Medicine (approved number: E1955). Written informed consent was obtained from each subject enrolled in this study.

### Blood pressure measurement

Home BPs were self-measured using a validated, automatic oscillometric device (Omron model HEM-7251G; Omron Corp., Tokyo, Japan). All recorded BP data were automatically transferred to a dedicated server. Participants were instructed to measure their home BP in the morning in a sitting position at rest. BP was measured within 1 hour after waking up, after urination, before breakfast, and before taking antihypertensive medication. BP was measured three times, and the average value was recorded. We computed the standard deviation (SD) of the systolic BP (SBP) recorded on seven consecutive days and the coefficient of variation (CV; SD divided by mean BP) as indicators of BP variability.

### VFA measurement

The HDS-2000 (Omron Healthcare, Kyoto, Japan) is a novel device for measurement of VFA using the dual bioelectrical impedance analysis (BIA) method [16–21]. Measurement using this device is carried out in the following procedure. First, the cross sectional area of the umbilical level abdomen is calculated from the anterior-posterior width and lateral width of the navel level abdomen measured with the attached measuring unit. Next, the lean body area is calculated from the impedance of the abdomen, which is generated when a weak current is made to flow from the extremity electrode. Further, the subcutaneous fat area is calculated from the abdominal impedance generated when a weak current flows between the electrodes of the abdomen. Finally, the visceral fat area is calculated from these measured values. The visceral fat area measured by this method has a good correlation with the value measured by X-ray CT [16][17][20]. This device provides accurate VFA measurements without X-ray exposure, and is reportedly an alternative to CT scanning. In the present study, we used VFA measured with the HDS-2000 as an indicator of fat accumulation. The height, weight, waist length and subcutaneous fat area (SFA) were measured simultaneously.

### Statistical analysis

To examine the relationship between VFA and BP variability, we divided the participants into three groups based on tertiles of VFA: low VFA, 23.6–69.5 cm2; intermediate VFA, 70.0–104.4 cm2; and high VFA, 105.9–181.6 cm2. To compare mean values among the three VFA groups, one-factor analysis of variance (ANOVA) was used. The tendency of the average value of the BP variability parameters of each group was evaluated using the Jonckheere-Terpstra test. Multiple linear regression analyses were also performed to examine the independent effects of the relationship between the VFA tertile and BP variability parameters, with adjustment for age, sex, mean BP level, diabetes mellitus, drinking habit (current habitual drinking) and the type of antihypertensive drug (renin-angiotensin-aldosterone system (RAAS) blocker, calcium channel blocker (CCB) and β-blocker). Values of two-tailed p<0.05 were considered significant. In addition, the same analyses were carried out for other obesity related indexes such as BMI and SFA, and they were compared with the result obtained for VFA. All statistical analyses were performed using EZR (Saitama Medical Center, Jichi Medical University, Saitama, Japan), which is a graphical user interface for R (The R Foundation for Statistical Computing, Vienna, Austria version 3.2.1) [22]. More precisely, it is a modified version of R commander designed to add statistical functions frequently used in biostatistics.

## Results

Table 1 summarizes the baseline characteristics and laboratory data of the study participants stratified based on their VFA tertiles. Overall, 41 participants (67.2%) were male with a mean age of 64.3 years, mean BMI of 24.5 kg/m2, and mean VFA of 88.0 cm2. Fifteen participants (24.6%) had diabetes mellitus, and 49 (80.3%) had dyslipidaemia. A total of 48 (78.7%) participants were treated with a RAAS blocker, including an angiotensin-converting enzyme (ACE) inhibitor, ARB, or mineralocorticoid receptor blocker. There were 34 (55.7%) participants taking a CCB and 17 (27.9%) taking a β-blocker. Participants in the higher VFA tertiles included more male patients and those with a higher body weight and BMI (all p<0.001).

**Table 1:** Baseline characteristics of participants stratified according to their VFA tertiles.

	Total (n = 61)	1st tertile VFA: 23.6–69.5 (n = 21)	2nd tertile VFA: 70.0–104.4 (n = 20)	3rd tertile VFA: 105.9–181.6 (n = 20)	p
Age, years, mean (SD)	64.3 (13.4)	67.6 (13.0)	60.8 (14.8)	64.4 (12.2)	0.28
Male, n (%)	41 (67.2)	8 (38.1)	14 (70.0)	19 (95.0)	<0.001
Body measurement, mean (SD)					
height, cm	162.3 (10.0)	156.9 (10.0)	163.5 (9.1)	166.8 (8.5)	0.004
BW, kg	65.0 (13.4)	53.9 (8.1)	64.4 (7.6)	77.4 (11.7)	<0.001
BMI, kg/m2	24.5 (3.6)	21.8 (2.3)	24.1 (2.4)	27.7 (3.2)	<0.001
waist, cm	85.9 (9.0)	78.9 (5.8)	84.4 (4.5)	95.8 (6.8)	<0.001
VFA, cm2	88.0 (34.6)	53.6 (13.1)	84.6 (11.4)	127.6 (22.4)	<0.001
SFA, cm^2^	171.3 (55.2)	136.9 (39.2)	162.3 (45)	216.5 (49.1)	<0.001
Laboratory data, mean (SD)					
T-Cho, mg/dL	198.8 (37.2)	194.7 (36.6)	208.7 (41.6)	192.5 (32.4)	0.34
LDL-Cho, mg/dL	108.6 (30.3)	106.9 (34.8)	115.8 (30.8)	101.2 (23.1)	0.39
HDL-Cho, mg/dL	59.0 (16.3)	61.7 (14.8)	61.3 (13.7)	53.9 (19.5)	0.25
Triglycerides, mg/dL	165.8 (102.9)	121.7 (65.2)	173.2 (85.3)	202.2 (134.4)	0.048
Casual Blood Glucose, mg/dL	105.6 (29.0)	107.5 (26.9)	99.2 (18.8)	110.2 (38.4)	0.48
HbA1c (NGSP), %	6.2 (0.8)	6.5 (0.9)	5.8 (0.8)	6.3 (0.6)	0.149
Habitual drinking, n (%)	38 (62.3)	11 (52.4)	13 (65.0)	14 (77.8)	0.26
Medication, n (%)					
RAAS blocker use	48 (78.7)	18 (85.7)	13 (65.0)	17 (85.0)	0.189
ACE-I use	6 (9.8)	3 (14.3)	1 (5.0)	2 (10.0)	0.61
ARB use	43 (70.5)	15 (71.4)	12 (60.0)	16 (80.0)	0.38
CCB use	34 (55.7)	9 (42.9)	13 (65.0)	12 (60.0)	0.32
thiazide use	12 (19.7)	3 (14.3)	2 (10.0)	7 (35.0)	0.103
alpha blocker use	2 (3.3)	0 (0.0)	1 (5.0)	1 (5.0)	0.58
beta blocker use	17 (27.9)	6 (28.6)	5 (25.0)	6 (30.0)	0.94
Medical History, n (%)					
Diabetes mellitus	15 (24.6)	5 (23.8)	4 (20.0)	6 (30.0)	0.76
Dyslipidemia	49 (80.3)	15 (71.4)	15 (75.0)	19 (95.0)	0.126
Chronic kidney disease	27 (44.3)	8 (38.1)	6 (30.0)	13 (65.0)	0.065
Cerebrovascular disease	9 (14.8)	2 (9.5)	6 (30.0)	1 (5.0)	0.059
ASO	2 (3.28)	2 (9.5)	0 (0.0)	0 (0.0)	0.140
hyperuricemia/ goat	13 (21.3)	1 (4.8)	5 (25.0)	7 (35.0)	0.054

BW, body weight; BMI, body mass index; VFA, visceral fat area; SFA, subcutaneous fat area; T-Cho, total cholesterol; LDL-cho, low-density lipoprotein cholesterol; HDL-Cho, high-density lipoprotein cholesterol; HbA1c, hemoglobin A1c; RAAS, renin-angiotensin aldosterone system; ACE-I, angiotensin-converting enzyme inhibitor; ARB, angiotensin receptor blocker; CCB, calcium channel blocker; ASO, arteriosclerosis obliterans.

**Fig 1:**
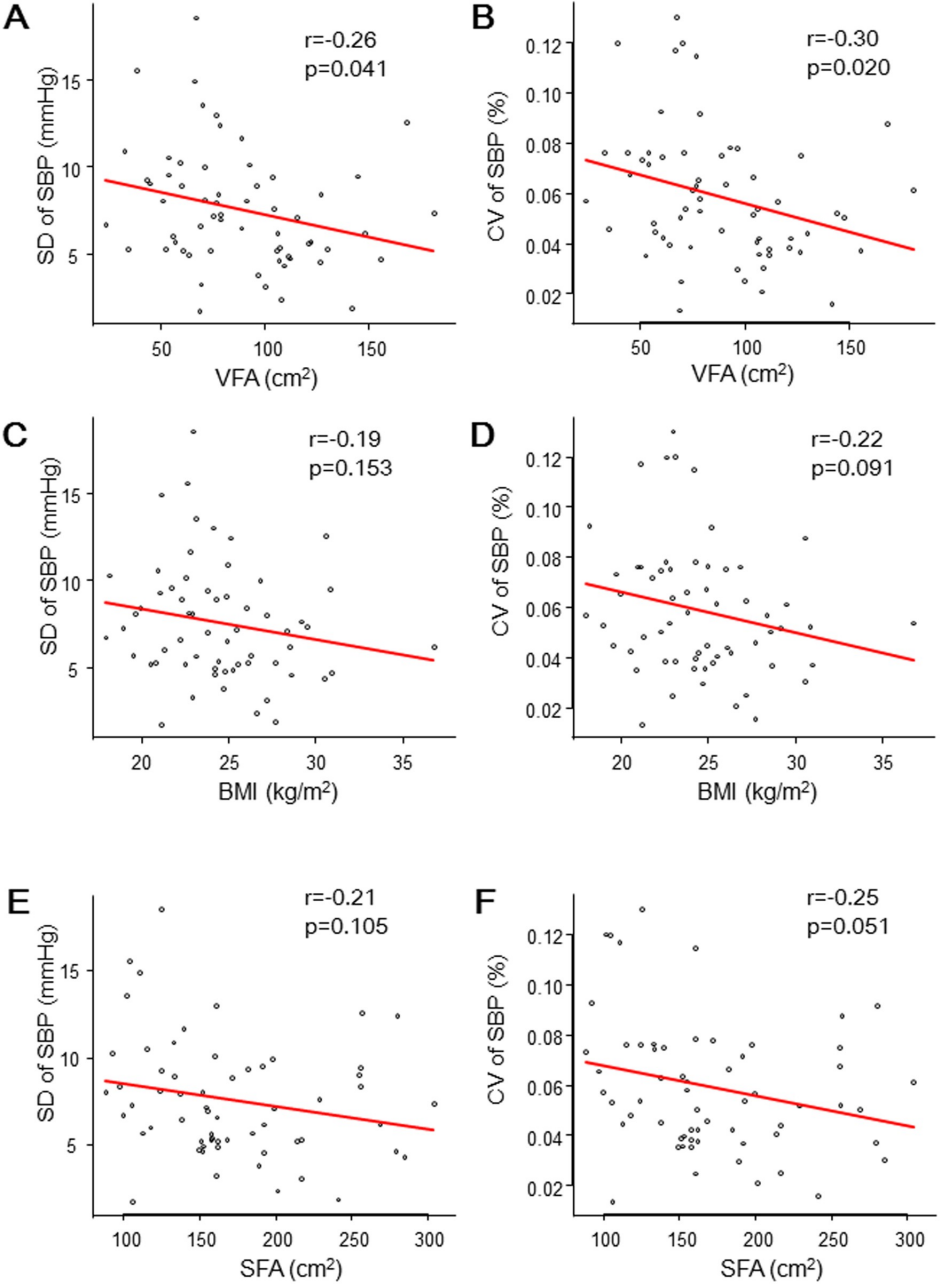
**(A) Correlation between SD of SBP and VFA area. (B) Correlation between CV of SBP and VFA area. (C) Correlation between SD of SBP and BMI. (D) Correlation between CV of SBP and BMI.** SBP, systolic blood pressure; VFA, visceral fat area; BMI, body mass index; SFA, subcutaneous fat area.

The absolute value and SD and CV of the home BPs and pulse rates are shown in Table 2. SBP levels were similar among all VFA tertiles, but the SD of the SBP was smaller in the larger VFA tertiles (p of ANOVA = 0.017, p for trend = 0.0126). Similarly, there was a negative correlation between VFA tertiles and the CV of the SBP (p of ANOVA = 0.012, p for trend = 0.0081). Multiple regression analysis adjusted by age, sex, antihypertensive drug (β-blocker, RAAS blocker, CCB), diabetes mellitus, drinking habit, and SBP level also showed significant association between VFA tertiles and the SD or CV of home SBP (Tables 3 and 4). The adjusted coefficient of regression for the SD was -3.03 (95%CI: -5.51 to -0.56, p = 0.017) and for the CV was -2.32 (95%CI: -4.23 to -0.41, p = 0.018) for the highest VFA group (3rd tertile) as compared to the lowest VFA group (1st tertile). Age, sex, type of anti-hypertensive drug, and presence of diabetes were not associated with the SD or CV of home SBP. We also performed a linear regression analysis using VFA as a continuous variable. VFA correlated negatively with the SD (p = 0.041) and CV (p = 0.020) of home BP. Similar analysis was performed using BMI or SFA instead of VFA, but no similar correlation was shown (Table 5, Fig 1).

**Table 2:** Absolute value and SD and CV of home BP and pulse rate.

	Total (n = 61)	1st tertile VFA: 23.6–69.5 (n = 21)	2nd tertile VFA: 70.0–104.4 (n = 20)	3rd tertile VFA: 105.9–181.6 (n = 20)	p
BP parameter, mean (SD)					
home SBP, mmHg	129.5 (12.7)	127.9 (10.4)	130.9 (12.6)	129.8 (15.4)	0.75
home DBP, mmHg	80.2 (9.7)	78.0 (9.8)	80.6 (10.1)	82.0 (9.1)	0.42
home PR, bpm	71.3 (10.4)	72.3 (13.0)	71.6 (9.3)	69.8 (8.6)	0.74
SD of SBP, mmHg	7.58 (3.39)	8.40 (4.15)	8.47 (2.80)	5.84 (2.37)	0.017
SD of DBP, mmHg	5.01 (2.54)	5.23 (2.92)	5.68 (2.40)	4.11 (2.05)	0.130
SD of PR, bpm	4.61 (2.54)	4.88 (3.36)	4.27 (2.28)	4.66 (1.79)	0.75
CV of SBP, %	5.88 (2.62)	6.57 (3.08)	6.55 (2.43)	4.48 (1.68)	0.012
CV of DBP, %	6.3 (3.25)	6.81 (3.86)	7.04 (2.96)	5.03 (2.5)	0.097
CV of PR, %	6.41 (3.28)	6.70 (4.27)	5.91 (3.01)	6.61 (2.32)	0.71

BP, blood pressure; SD, SBP, systolic blood pressure; DBP, diastolic blood pressure; PR, pulse rate

**Table 3:** Simple correlation and multiple regression analysis of the standard deviation of home BP.

Variable	SD of Home SBP			
	Univariate		Multivariate	
	β (SE)	P	β (SE)	p
Age, year	-0.00 (0.03)	0.96	-0.03 (0.04)	0.50
Sex (men = 0, women = 1)	0.51 (0.93)	0.59	-1.20 (1.10)	0.28
Home SBP, mmHg	0.04 (0.03)	0.24	0.05 (0.05)	0.25
VFA tertile				
2nd tertile (as compared to 1st tertile)	0.06 (1.00)	0.95	-0.21 (1.15)	0.86
3rd tertile (as compared to 1st tertile)	-2.57 (1.00)	0.013	-3.03 (1.22)	0.017
beta blocker use, (no = 0, yes = 1)	0.19 (0.98)	0.85	0.79 (1.00)	0.43
CCB use, (no = 0, yes = 1)	0.08 (0.88)	0.93	-0.23 (1.03)	0.83
RAAS blocker use, (no = 0, yes = 1)	0.31 (1.07)	0.77	0.74 (1.24)	0.55
Diabetes, (no = 0, yes = 1)	0.76 (1.01)	0.46	1.24 (1.09)	0.26
Habitual drinking (no = 0, yes = 1)	-1.54 (0.90)	0.092	-1.33 (1.09)	0.23

Multiple R-squared: 0.2175, Adjusted R-squared: 0.05442

**Table 4:** Simple correlation and multiple regression analysis of the coefficient of variation of home BP.

Variable	CV of Home SBP			
	Univariate		Multivariate	
	β (SE)	P	β (SE)	p
Age, year	0.00 (0.03)	0.96	-0.02 (0.03)	0.58
Sex (men = 0, women = 1)	0.47 (0.72)	0.52	-0.88 (0.85)	0.31
Home SBP, mmHg	-0.02 (0.03)	0.54	-0.01 (0.03)	0.76
VFA tertile				
2nd tertile (as compared to 1st tertile)	-0.02 (0.77)	0.98	-0.02 (0.89)	0.98
3rd tertile (as compared to 1st tertile)	-2.09 (0.77)	0.009	-2.32 (0.95)	0.018
beta blocker use, (no = 0, yes = 1)	0.43 (0.75)	0.57	0.62 (0.77)	0.43
CCB use, (no = 0, yes = 1)	0.07 (0.68)	0.91	-0.17 (0.80)	0.84
RAAS blocker use, (no = 0, yes = 1)	0.35 (0.83)	0.68	0.59 (0.96)	0.54
Diabetes, (no = 0, yes = 1)	1.04 (0.78)	0.187	1.00 (0.85)	0.24
Habitual drinking (no = 0, yes = 1)	-1.24 (0.70)	0.08	-1.05 (0.84)	0.22

Multiple R-squared: 0.2257, Adjusted R-squared: 0.06438

**Table 5:** Univariate and multivariate linear regression analysis of obesity indices as a continuous variable with SD and CV of home SBP.

Variable	SD of Home SBP			
	Univariate		Multivariate*	
	β (SE)	p	β (SE)	p
VFA, cm^2^	-0.03 (0.01)	0.041	-0.04 (0.02)	0.023
SFA, cm^2^	-0.01 (0.01)	0.105	-0.01 (0.01)	0.125
BMI, kg/m^2^	-0.17 (0.12)	0.153	-0.14 (0.14)	0.30
Variable	CV of Home SBP			
	Univariate		Multivariate*	
	β (SE)	p	β (SE)	p
VFA, cm^2^	-0.02 (0.01)	0.020	-0.03 (0.01)	0.021
SFA, cm^2^	-0.01 (0.01)	0.051	-0.01 (0.01)	0.106
BMI, kg/m^2^	-0.16 (0.09)	0.090	-0.12 (0.11)	0.26

SBP, systolic blood pressure; VFA, visceral fat area; SFA, subcutaneous fat area; BMI, body mass index

*These models are adjusted for age, sex, antihypertensive drug (β-blocker, RAAS blocker, CCB), diabetes mellitus, habitual drinking, and SBP level

## Discussions

This cross-sectional study is the first to examine the correlation between the day-to-day variability of home BP and VFA measured using the dual-scan method. In this study, VFA area was significantly associated with day-to-day variability in home BP in 61 adult hypertensive patients.

Several reports have shown that day-to-day variability in home BP may be an independent prognostic factor for cardiovascular risk [6–9]. Therefore, clarifying factors affecting the variability of home BP could provide useful information for assessing cardiovascular risk and potential therapeutic targets. Indeed, it has been reported that age, sex, drinking habits, sleep time and walking time as well as antihypertensive drug use, pulse rate, and BMI are all associated with day-to-day BP variability [9,11,23,24].

Visceral fat accumulation is considered to be a risk factor for arteriosclerosis, while day-to-day variation in home BP is a reflection of arterial stiffness [25]. We therefore hypothesized that these two factors would be positively correlated. Instead, the present study showed a negative correlation between visceral fat accumulation and day-to-day home BP variability. Similarly an earlier study reported a negative correlation between BMI and day-to-day home BP variability [11]. Because VFA reflects the state of obesity as well as BMI, it is reasonable that VFA correlates negatively with BP variability. However, in this study, there was no significant correlation between BMI and BP variability. This may be because VFA more acutely reflects the pathophysiological state of obesity than BMI, especially in the Japanese. Asian populations reportedly have different associations between BMI, percent body fat, and health risks than do European populations [12,13]. VFA is larger in the Japanese than Caucasians after adjusting subcutaneous fat area, age and sex [13], and the average BMI in Japanese diabetic patients (23.1 kg/m2) is significantly lower than that in patients in UK (29.4 kg/m2) [12]. The number of subjects in this study may have been insufficient in detecting power to detect a significant association between BMI and BPV, but it was enough to detect the association between VFA and BPV.

The likely relationship between BMI or VFA and BP variability reported previously and in the present study suggests a possible link between the pathophysiological conditions associated with obesity-related hypertension and a reduction in home BP variability. Major pathological conditions associated with obesity include 1) sympathetic nerve system activation, 2) RAAS activation, and 3) sodium retension which leads to fluid retention [10,26–28]. In an interindividual assessment of visit-to-visit BP variability, β-blocker usage was associated with higher BP variability [29]. Moreover, the same study reported that ARB usage is also associated with higher BP variability [29]. And in another report, use of an ACE inhibitor was associated with higher BP variability than use of a CCB or diuretic [30]. Nonetheless, one trial that compared between CCB and diuretics showed that diuretics usage was associated with higher BP variability [25]. Thus sympathetic nervous system blockade, RAAS blockade and accelerated natriuretic state are all associated with higher BP variability. These findings suggest that the aforementioned pathological conditions associated with visceral fat accumulation can lead to reductions in the variability of home BP.

This negative correlation could also be interpreted from the viewpoint of the time course of the pathological process, in which fat accumulation precedes the development of arteriosclerosis. Future longitudinal follow-up will be necessary to fully understand the observed correlation. Nevertheless, this study provides a novel finding, that there is a negative correlation between visceral fat area and day-to-day home BP variability, which contributes to our understanding of the pathological association between the obesity and atherosclerotic diseases.

The clinical significance of this study is to show that the degree of obesity should be taken into account when evaluating the value of BPV. Further research on the possibility of involvement of sympathetic nerve, RAAS activity, circulating plasma volume is expected for the mechanism of correlation between obesity and BPV.

## Limitations

There are three main limitations to this study. First, we could not fully correct for factors that could potentially influence home BP variability, such as adherence to an antihypertensive drug, lifestyle factors such as walking time and sleeping time. Second, this study was cross-sectional and could not evaluate the causal relationship between VFA and variability in home BP. To clarify the causal relationship, a longitudinal study and, perhaps, an interventional study are needed. Thirdly, the sample size of this study was relatively small. The reason why correlation with BPV could not be detected by factors other than VFA may be due to the small sample size. On the other hand, the association between VFA and BPV was sufficiently strong and it was possible to detect even with this small sample size.

## Conclusions

In conclusion, our results show for the first time that there is a negative correlation between day-to-day variability of home blood pressure and visceral fat area in hypertensive patients. Future research to clarify its mechanism and establish clinical significance of blood pressure variability and visceral fat measurement is desired.

## Supporting information

S1 Dataset List of all the 61 participants data analyzed in the present study. (CSV)
